# Endometrial stromal sarcomas with *BCOR*‐rearrangement harbor *MDM2* amplifications

**DOI:** 10.1002/cjp2.165

**Published:** 2020-04-30

**Authors:** Felix KF Kommoss, Kenneth TE Chang, Damian Stichel, Ana Banito, David TW Jones, Christoph E Heilig, Stefan Fröhling, Felix Sahm, Albrecht Stenzinger, Wolfgang Hartmann, Gunhild Mechtersheimer, Hans‐Peter Sinn, Dietmar Schmidt, Friedrich Kommoss, Andreas von Deimling, Christian Koelsche

**Affiliations:** ^1^ Department of Pathology, Institute of Pathology Heidelberg University Hospital Heidelberg Germany; ^2^ Department of Pathology and Laboratory Medicine KK Women's and Children's Hospital Singapore Singapore; ^3^ Department of Neuropathology, Institute of Pathology Heidelberg University Hospital Heidelberg Germany; ^4^ Clinical Cooperation Unit Neuropathology German Cancer Consortium (DKTK), German Cancer Research Center (DKFZ) Heidelberg Germany; ^5^ Hopp Children's Cancer Center Heidelberg (KiTZ) Heidelberg Germany; ^6^ Pediatric Soft Tissue Sarcoma Research Group German Cancer Research Center (DKFZ) Heidelberg Germany; ^7^ Pediatric Glioma Research Group, German Cancer Consortium (DKTK) and German Cancer Research Center (DKFZ) Heidelberg Germany; ^8^ German Cancer Consortium (DKTK) Heidelberg Germany; ^9^ Division of Translational Medical Oncology DKFZ and NCT Heidelberg Heidelberg Germany; ^10^ Division of Translational Pathology, Gerhard Domagk Institute of Pathology University Hospital Münster Münster Germany; ^11^ MVZ für Histologie Zytologie und Molekulare Diagnostik Trier GmbH Trier Germany; ^12^ Institute of Pathology Medizin Campus Bodensee Friedrichshafen Germany

**Keywords:** endometrial stromal sarcoma, MDM2, amplification, BCOR, YWHAE, uterine, neoplasm

## Abstract

Recently a novel subtype of endometrial stromal sarcoma (ESS) defined by recurrent genomic alterations involving *BCOR* has been described (HGESS‐BCOR). We identified a case of HGESS‐BCOR with a *ZC3H7B‐BCOR* gene fusion, which harbored an amplification of the *MDM2* locus. This index case prompted us to investigate *MDM2* amplification in four additional cases of HGESS‐BCOR. Tumors were analyzed for *MDM2* amplification by array‐based profiling of copy number alterations (CNAs) and fluorescence *in situ* hybridization (FISH), as well as for MDM2 expression by immunohistochemistry (IHC). Additionally, a cohort of other mesenchymal uterine neoplasms, including 17 low‐grade ESS, 6 classical high‐grade ESS with *YWHAE*‐rearrangement, 16 uterine tumors resembling ovarian sex cord tumors, 7 uterine leiomyomas and 8 uterine leiomyosarcomas, was analyzed for CNAs in *MDM2*. Copy number profiling identified amplification of the 12q15 region involving the *MDM2* locus in all five HGESS‐BCOR. Subsequent validation analyses of three tumors confirmed *MDM2* amplification using *MDM2* FISH. Accordingly, IHC showed MDM2 overexpression in all analyzed cases. None of the other uterine neoplasms in our series, including tumors that are in the histopathological differential diagnoses of HGESS‐BCOR, showed copy number gains of *MDM2*. Together, our results indicate that HGESS‐BCOR carries *MDM2* amplifications, which has diagnostic implications and could potentially be used for targeted therapies in these clinically aggressive tumors.

## Introduction

Endometrial stromal tumors (EST) represent rare uterine neoplasms of mesenchymal origin [1]. The 2014 WHO classification of tumors of the female reproductive tract distinguishes four categories of EST: endometrial stromal nodule (ESN), low‐grade endometrial stromal sarcoma (LGESS), high‐grade endometrial stromal sarcoma (HGESS), and undifferentiated uterine sarcoma (UUS) [2]. While ESN and LGESS histologically resemble stromal cells of the proliferating endometrium and harbor recurrent chromosomal translocations most frequently associated with a *JAZF1‐SUZ12* gene fusion, UUS comprises endometrial and myometrial sarcomas which lack specific mesenchymal differentiation and are molecularly heterogenous [3, 4]. As defined by the 2014 WHO classification, HGESS harbor a t(10;17)(q22;p13) chromosomal translocation resulting in a *YWHAE‐NUTM2* fusion [2, 5]. Such tumors represent a clinically more aggressive entity with patients diagnosed at higher stages and more likely to die of disease when compared to LGESS [6].

Recently, a rare subtype of ESS with high‐grade features and *BCOR* alterations, caused by either a gene fusion between *BCOR* and *ZC3H7B* or a mutually exclusive somatic internal tandem duplication (ITD) of exon 15 of *BCOR*, has been described (HGESS‐BCOR) [7, 8]. Although such *BCOR* alterations may well be the molecular driver in these tumors, little is known about their biology, or about potential cooperative and co‐occurring genetic events.

We recently identified a case of HGESS‐BCOR that carried an amplification of the 12q15 region involving the *MDM2* locus. This observation prompted us to compile a multicenter cohort to investigate *MDM2* amplification in HGESS‐BCOR.

## Material and methods

### Study cohort

A study cohort including HGESS‐BCOR, LGESS, HGESS, uterine tumors resembling ovarian sex cord tumors (UTROSCT), uterine leiomyomas (ULMO), and uterine leiomyosarcomas (ULMS) was collected from the referral center archives of two of the authors (DS and FK), the Department of Pathology, University of Heidelberg, and the KK Women's and Children's Hospital, Singapore. All cases were subject to expert pathology review including molecular pathology [9]. Fusion status of HGESS‐BCOR has previously been reported in part [10, 11]. This study was performed in accordance with the ethical standards of the institutional research committee and the Declaration of Helsinki.

### Genomic DNA extraction and quantification

DNA of all tumors was extracted from formalin‐fixed paraffin‐embedded (FFPE) tissue samples. Extracted DNA was quantified using the QuantiFast SYBR Green PCR Kit (Qiagen, Duesseldorf, NW, Germany).

### Copy number‐profile generation

A total of >100 ng DNA was available for array‐based DNA methylation analysis in all cases. Samples were analyzed using the Illumina Infinium HumanMethylation450 (450k) or EPIC (850k) BeadChip (Illumina, San Diego, IL, USA), according to the manufacturer's instructions at the Genomics and Proteomics Core Facility of the German Cancer Research Center (DKFZ), Heidelberg. DNA methylation data were normalized by performing background correction and dye bias correction as previously described [11]. Probes targeting sex chromosomes, probes containing multiple single nucleotide polymorphisms, and those which could not be uniquely mapped, were removed.

### *MDM2*‐specific fluorescence *in situ* hybridization

*MDM2*‐specific fluorescence *in situ* hybridization (FISH) analysis was performed on whole tissue sections using the ZytoLight® SPEC MDM2/CEN 12 Dual Color Probe (ZytoVision GmbH, Bremerhaven, Germany) as previously described [12]. Amplification of *MDM2* was defined as an *MDM2*/centromere 12 (CEN12) ratio ≥2.0 or an average number of *MDM2* signals per tumor cell nucleus ≥6 or large clusters of MDM2 signals in ≥10% of tumor cells.

### MDM2 immunohistochemistry

Four micrometer sections were cut and mounted on StarFrost Advanced Adhesive slides (Engelbrecht, Kassel, Germany) followed by heat induced antigen retrieval in high pH buffer. MDM2 immunohistochemistry was performed using a monoclonal mouse antibody (dilution 1:100, clone IF2, Invitrogen by Thermo Fisher Scientific Inc., Waltham, MA, USA) as previously described [12]. Specimens were examined according to their nuclear staining for MDM2.

## Results

### Clinicopathologic characteristics of HGESS‐BCOR

**Figure 1:**
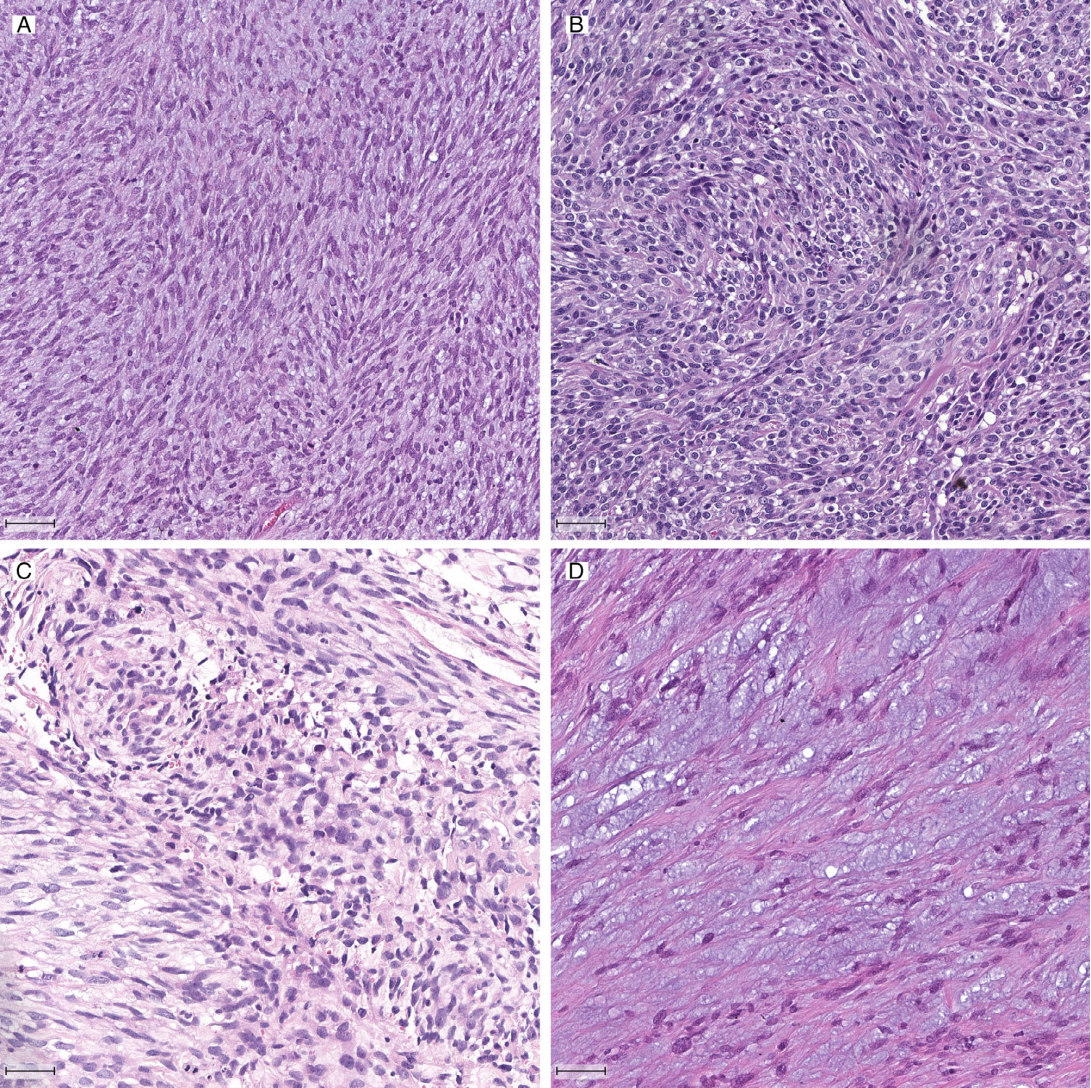
Histologic features of HGESS‐BCOR: sarcomatous proliferation of atypical and spindled neoplastic tumor cells with fascicular architecture (A and B), notable atypia (C) and prominent myxoid stroma (D). H&E stain; bar equals 50 μm.

Five HGESS‐BCOR were available for analysis. All tumors showed a hypercellular appearance with haphazard fascicular architecture. Tumor cells were spindled with irregular nuclear contours and an even chromatin pattern (Figure 1A,B). More notable atypia was only seen in one tumor (case 2; Figure 1C). Island‐like myxoid stromal change was present in three tumors (cases 1, 3, and 4; Figure 1D). RNA‐seq analysis identified a *ZC3H7B‐BCOR* gene fusion in four cases (cases 1, 2, 4, and 5). In case 3, the fusion detection algorithm identified a rearrangement between *BCOR* and the *LPP* gene, including RNA reads spanning parts of both genes. This event, however, appears to result in overexpression of a C‐terminally truncated BCOR protein, without generation of a fusion protein (no coding part of LPP is included). Thus, while RNA‐seq data support the presence of a *BCOR* alteration, the precise functional consequences of this aberration remain unclear. Clinicopathological data of all HGESS‐BCOR cases are summarized in Table 1.

**Table 1:** Clinicopathological data of HGESS‐BCOR cases

Case ID	Age	Diagnosis	Specimen type	Myxoid change	Molecular type
Case 1	48	HGESS‐BCOR	Hysterectomy	Yes	*ZC3H7B‐BCOR*
Case 2	31	HGESS‐BCOR	Hysterectomy	No	*ZC3H7B‐BCOR*
Case 3	50	HGESS‐BCOR	Hysterectomy	Yes	*BCOR* alteration
Case 4	41	HGESS‐BCOR	Hysterectomy	Yes	*ZC3H7B‐BCOR*
Case 5	34	HGESS‐BCOR	Hysterectomy	No	*ZC3H7B‐BCOR*

### *MDM2* amplification in HGESS‐BCOR

**Figure 2:**
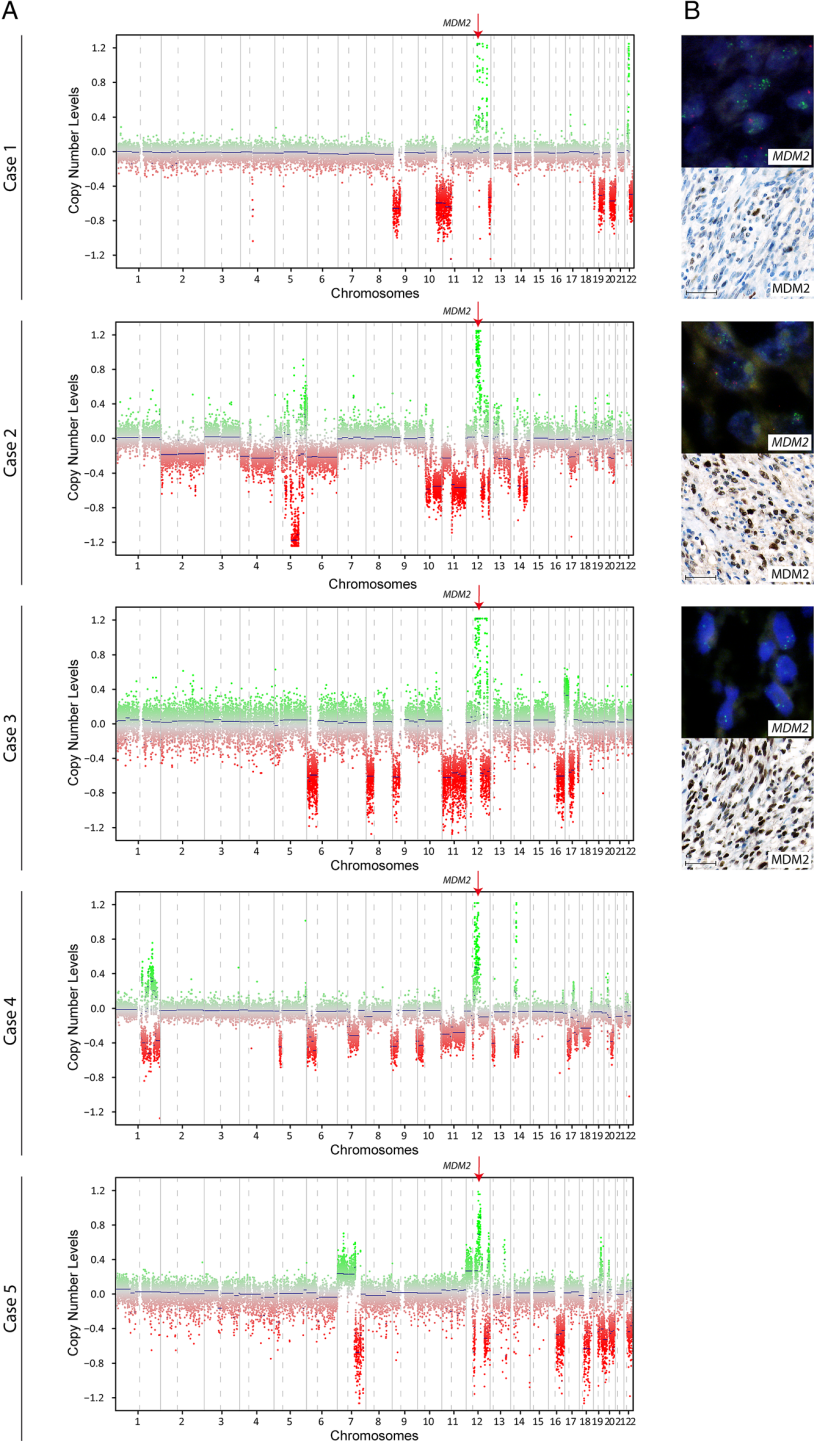
*MDM2* amplification in HGESS‐BCOR: (A) copy number profiles (cases 1–5). (B) *MDM2* FISH (MDM2: green, CEN12: red) and immunohistochemical staining for MDM2 (cases 1–3). Bar equals 50 μm.

Copy number analysis of HGESS‐BCOR showed an amplification of the 12q15 region involving the *MDM2* locus in all five cases (Figure 2A). For subsequent MDM2 validation analyses sufficient material was only available for three tumors (cases 1–3). FISH and IHC confirmed the amplification of *MDM2* and overexpression (Figure 2B) of MDM2 in all three tumors. *MDM2* amplification by FISH was present diffusely across the tumors analyzed. In addition to an *MDM2* amplification, copy number analyses also revealed amplification of *CDK4* in three tumors (cases 1–3) and a deletion of *CDKN2A* in one tumor (case 5).

### *MDM2* amplification is rare in other mesenchymal uterine neoplasms

Next, we analyzed copy number profiles of potential differential diagnoses of HGESS‐BCOR including 17 LGESS, 6 HGESS with *YWHAE‐NUTM2* gene fusion, 16 UTROSCT, 7 ULMO, and 8 ULMS. None of these cases carried an amplification of the 12q15 region involving the *MDM2* locus (Table 2).

**Table 2:** 12q15 (*MDM2*) amplification status of mesenchymal uterine neoplasms including LGESS, HGESS‐BCOR, HGESS with *YWHAE*‐rearrangement, UTROSCT, ULM, and ULMS

Diagnosis	12q15 amplification (*MDM2*)
Low‐grade endometrial stromal sarcoma	0/17 (0%)
High‐grade endometrial stromal sarcoma (*BCOR*)	5/5 (100%)
High‐grade endometrial stromal sarcoma (*YWHAE‐NUTM2*)	0/6 (0%)
Uterine tumor resembling ovarian sex cord tumor	0/15 (0%)
Uterine leiomyoma	0/8 (0%)
Uterine leiomyosarcoma	0/7 (0%)

## Discussion

HGESS‐BCOR represents a new subtype in the spectrum of EST, defined by genetic alterations involving *BCOR*. ESS with *BCOR* alteration show a greater degree of atypia when compared to LGESS, and available clinical data suggest an aggressive clinical course similar to that of classical HGESS [13]. Thus, it has been proposed to place such tumors in the HGESS category. We have recently shown *BCOR*‐ and *YWHAE*‐rearranged HGESS to share similar DNA methylation profiles, distinct from LGESS and other high‐grade uterine sarcomas such as ULMS, further supporting the proposed classification [11]. In the current study, we show HGESS‐BCOR to harbor *MDM2* amplifications, while all HGESS with *YWHAE‐NUTM2* gene fusion in our series were *MDM2*‐balanced.

MDM2 is the primary negative regulator of p53 and is overexpressed in cancers, *e.g*. certain subtypes of sarcomas [14]. Mechanistically, MDM2 overexpression functions as a powerful oncogene by negatively regulating *TP53* transcriptional activity and therefore has been suggested as a druggable target for small molecule inhibitors [15]. Given the above, p53 immunohistochemistry in a subset of HGESS‐BCOR of our cohort did not exhibit strong or diffuse p53 expression (*n* = 3). In line with this staining pattern, additional DNA sequencing did not identify pathogenic *TP53* mutations in any HGESS‐BCOR of our series (*n* = 5; data not shown). Thus, detecting *MDM2* amplification in HGESS‐BCOR might have clinical implications in affected patients. To date, therapeutic strategies in cases of high‐grade uterine sarcoma are usually limited to surgery and radio‐/chemotherapy, and prognosis is generally dismal [16]. Targeting MDM2 might therefore be a promising way to extend the spectrum of therapeutic options for this aggressive neoplasm.

MDM2 has previously been investigated in EST (*n* = 43) including ESN, LGESS, HGESS with *YWHAE*‐rearrangement, and UUS [17]. Schoolmeester *et al* reported *MDM2* amplification in two of 43 (5%) tumors, both of which showed an adverse clinical course. One of the latter tumors showed morphological features consistent with LGESS, and it was reported to harbor a *JAZF1‐*rearrangement. The second tumor harboring a *MDM2* amplification had high‐grade morphology, polysomy of *JAZF1*, *PHF1*, and *YWHAE* but no rearrangements were reported, leading to a diagnosis of UUS. While the latter data imply that *MDM2* amplification is also present in a subset of LGESS and UUS, we did not identify copy number gains of *MDM2* in any other mesenchymal uterine tumor of our series including 17 LGESS. Interestingly, a recent study suggests that UUS often represents under‐recognized HGESS including tumors with *BCOR* alteration [18]. Moreover, ESS may rarely present as a primary intrabdominal soft tissue tumor or arise at distant sites in extragenital endometriosis. This is important to know when encountering undifferentiated mesenteric or retroperitoneal soft tissue tumors. While *MDM2* amplification in such tumors usually supports a diagnosis of dedifferentiated liposarcoma, HGESS‐BCOR should also be considered in the differential diagnoses in such cases [19]. Notably, MDM2 positivity by immunohistochemistry has been reported in cases of uterine leiomyosarcoma and primary liposarcoma of the uterus [20, 21]. However, to date no systematic analysis of *MDM2* amplification has been performed in such cases.

As a limitation of our study, it is important to mention that our study cohort did not include cases of Müllerian adenosarcoma, a lesion known to harbor *MDM2* amplifications in up to 28% of cases [22]. Further, our study did not investigate tumors with *BCOR‐*ITD, which are exceedingly rare with only few cases reported in the literature to date [8]. Thus, future studies of larger cohorts of mesenchymal uterine neoplasms are needed to confirm, and to expand on, our results.

In conclusion, our data suggest that detection of *MDM2* amplicons could be a useful tool in uterine sarcoma pathology and implicate MDM2 as a potential therapeutic target in HGESS‐BCOR.

## Author contributions statement

FKFK and CK conceptualized the project and wrote the original draft. FKFK and CK coordinated data generation. FS and AvD supervised array‐based analysis. DSt, DTWJ, and AB analyzed array data. WH supervised the FISH and IHC analysis. FKFK, KTC, CEH, SF, AS, GM, HPS, DS, and FK provided tumor samples and corresponding metadata. All authors contributed to and approved of the final manuscript.
